# The APC-EPCR-PAR1 axis in sickle cell disease

**DOI:** 10.3389/fmed.2023.1141020

**Published:** 2023-07-11

**Authors:** Nirupama Ramadas, Erica M. Sparkenbaugh

**Affiliations:** ^1^Department of Medicine, Blood Research Center, University of North Carolina at Chapel Hill, Chapel Hill, NC, United States; ^2^Department of Pathology and Laboratory Medicine, University of North Carolina at Chapel Hill, Chapel Hill, NC, United States

**Keywords:** sickle cell disease, vaso-occlusion, inflammation, sickle cell anemia, protease activated receptor 1 (PAR1), endothelial protein C receptor (EPCR), activated protein C (APC), thrombin

## Abstract

Sickle Cell Disease (SCD) is a group of inherited hemoglobinopathies. Sickle cell anemia (SCA) is caused by a homozygous mutation in the β-globin generating sickle hemoglobin (HbS). Deoxygenation leads to pathologic polymerization of HbS and sickling of erythrocytes. The two predominant pathologies of SCD are hemolytic anemia and vaso-occlusive episodes (VOE), along with sequelae of complications including acute chest syndrome, hepatopathy, nephropathy, pulmonary hypertension, venous thromboembolism, and stroke. SCD is associated with endothelial activation due to the release of danger-associated molecular patterns (DAMPs) such as heme, recurrent ischemia–reperfusion injury, and chronic thrombin generation and inflammation. Endothelial cell activation is mediated, in part, by thrombin-dependent activation of protease-activated receptor 1 (PAR1), a G protein coupled receptor that plays a role in platelet activation, endothelial permeability, inflammation, and cytotoxicity. PAR1 can also be activated by activated protein C (APC), which promotes endothelial barrier protection and cytoprotective signaling. Notably, the APC system is dysregulated in SCD. This mini-review will discuss activation of PAR1 by APC and thrombin, the APC-EPCR-PAR1 axis, and their potential roles in SCD.

## Introduction

### Sickle cell disease

**Figure 1:**
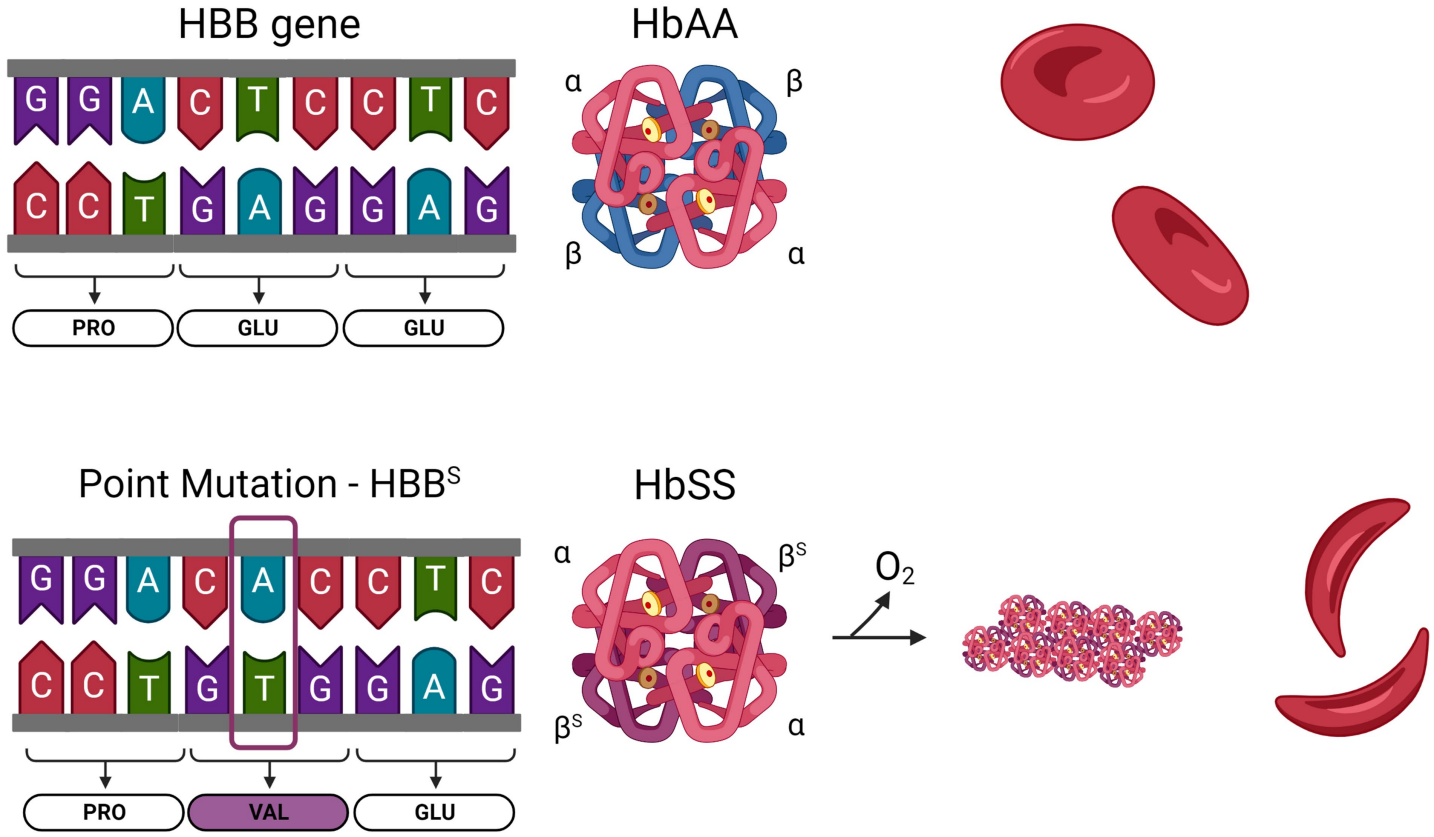
Sickle cell anemia is caused by a single nucleotide mutation in the *HBB* gene. Normal hemoglobin (HbAA) is formed from two α-globin and two β-globin subunits. The HBB gene encodes β-globin. In SCD, an adenine to thymine substitution changes the 6th codon of the mature protein from a glutamine to a valine. Sickle hemoglobin (HbSS) contains two α-globin and two β^S^-globin. Upon deoxygenation, the presence of a hydrophobic valine residue in HbSS causes the molecules to polymerize, leading to stiffening of the RBC.

Sickle cell disease (SCD) is the most common inherited hemoglobinopathy worldwide. More than 300,000 babies are born with SCD annually and this rate is expected to increase over the next 30 years (1). The majority of cases are concentrated in sub-Saharan Africa and southern Asia, where sickle cell trait provides protection from malaria (2). Sickle Cell Anemia (SCA) is caused by a single nucleotide mutation in the gene for beta (β) globin. Normal hemoglobin is a tetramer of two α and two β subunits, each of which contain a heme molecule, and is the critical oxygen carrying protein in red blood cells (RBCs). In SCA, an A to T transversion in the sixth codon results in the substitution of a valine (Val) for a glutamine (Glu). This hydrophobic valine residue confers an adhesive property to HbS (comprised of two α and two β^S^ subunits); thus, when it becomes deoxygenated it forms rigid polymers in RBCs (Figure 1). This results in the sickling of RBCs, causing hemolysis and anemia. Hemolysis releases HbS and free heme into the circulation, acting as danger associated molecular patterns (DAMPs) to activate the endothelium and leukocytes. The activated endothelium upregulates adhesion molecules E-selectin, P-selectin, vascular cell adhesion molecule (VCAM), intercellular adhesion molecule (ICAM), and von Willebrand Factor (VWF). Activated neutrophils, platelets, and sickled RBCs form multicellular aggregates in the circulation, which adhere to the circulating endothelium, leading to vaso-occlusive episodes (VOE). These two primary pathologies, hemolytic anemia and VOE, are accompanied by sequelae of acute and chronic complications such as acute painful crises (3), chronic pain, stroke (4–8), venous thrombosis and pulmonary embolism (9, 10), pulmonary hypertension (11), acute chest syndrome (12), sickle nephropathy (13, 14), among others. More comprehensive reviews of SCD and its complications can be found elsewhere (3, 15).

### Current treatments for SCD

Despite the significant global burden of SCA, there are only four FDA-approved drugs currently available to patients: hydroxyurea, L-glutamine, crizanlizumab, and Voxelotor (16). Hydroxyurea inhibits HbS polymerization and sickling by increasing production of fetal Hb (HbF) (17). L-glutamine reduces oxidative stress (18). Crizanlizumab inhibits P-selectin-dependent sRBC-endothelial interactions (19). Voxelotor changes the affinity of HbS for oxygen and inhibits hemoglobin polymerization (20). These therapies modestly limit the severity and frequency of VOC (17–19). Many SCA patients also routinely undergo whole blood transfusions and red blood cell exchange therapy (3, 21). Curative options such as hematopoietic stem cell transplantation (HSCT) and gene therapy (GT) are also being investigated (22). Allogeneic HSCT has been performed in approximately 2,000 patients in the past 30 years. A recent meta-analysis revealed that HSCT reduces the incidence of VOE, but it also identified risks including graft-versus host disease, graft failure, mortality, and secondary malignancies (23). The GT strategies currently being evaluated are correction of the HbS mutation, gene transfer to overexpress HbA in hematopoietic stem cells, and knockdown of BCL11a, the negative regulator of HbF, to increase HbF production and prevent sickling (3, 22–24). Although these therapies are promising, the FDA may require long-term follow up of 10–15 years after gene therapy to evaluate safety risks before they will be approved for clinical use (25). HSCT and GT carry significant costs and medical resources, and may not be feasible in low-resource countries where SCA is most prevalent (22). Recent GT trials have also been paused due to unexpected toxicity (26). The limited range of approved drugs for SCA, combined with an ageing population facing severe clinical complications, highlights the need to investigate new treatment options that are accessible and effective. Several drugs targeting downstream events are currently being evaluated in Phase II and III clinical trials, including anti-sickling agents, anti-inflammatory agents, and anticoagulants (3, 15).

### Coagulation activation in SCA

A hallmark of SCA is activation of coagulation (27–33). Tissue factor (TF) is the primary initiator of extrinsic coagulation and is not normally expressed on intravascular cells. In SCA, TF expression is upregulated on leukocytes and endothelial cells (34–37). TF is a transmembrane protein and obligate cofactor for coagulation factor VIIa (FVIIa), activating factor X (FX) to FXa, which converts prothrombin to thrombin. Thrombin cleaves fibrinogen into fibrin, leading to clot formation. We and others have shown that TF (4, 5, 37, 38), FXa (6, 39), thrombin (39, 40), and fibrin(ogen) (41, 42) contribute to inflammation, cardiovascular dysfunction, vascular congestion, nephropathy, and microvascular stasis (43) in mouse models of SCA. In addition to its prothrombotic role, thrombin can induce signaling through Protease Activated Receptor-1 (PAR1).

### Protease activated receptors

**Figure 2:**
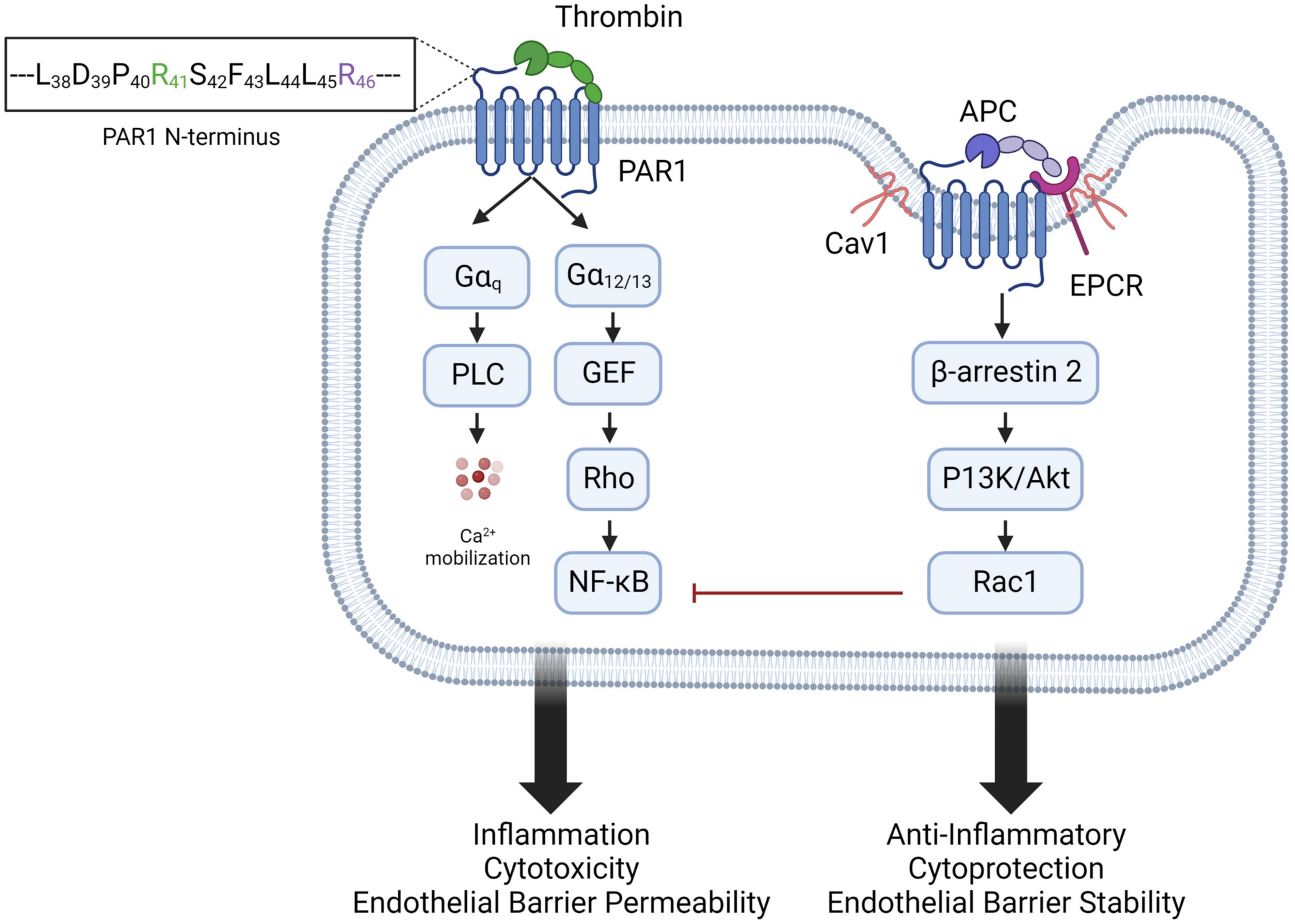
Biased agonism of PAR1 by thrombin and APC. Thrombin activation of PAR1, by cleavage at arginine 41 (R41) favors activation of Gαq and Gα12/13 signaling pathways. This results in inflammation, endothelial activation and barrier permeability, and cytotoxicity. In caveolin 1 (Cav1)-positive lipid rafts, PAR1 colocalizes with endothelial protein C receptor (EPCR). Activated protein C (APC) binds EPCR and activates PAR1 by cleavage of arginine 46 (R46), recruiting β-arrestin and inducing anti-inflammatory and endothelial stabilizing signaling.

Protease activated receptors (PARs) are a family of G-protein coupled receptors (GPCR) consisting of PAR1, PAR2, PAR3, and PAR4. PARs share a conserved mechanism of irreversible activation by proteolytic cleavage of specific amino acid residues on the extracellular N-terminus. This results in the exposure of a novel N-terminal peptide, or tethered ligand, which binds to extracellular loop 2 and induces a conformational change in the GPCR to signal through intracellular G proteins (44). PARs are subject to proteolysis by multiple proteases at different amino acid residues on the N-terminus, resulting in activation of different signaling pathways and outputs (45). The focus of this review is PAR1, which was first identified as the main thrombin receptor on platelets (46–48), triggering activation and aggregation of platelets that is critical for both hemostasis and thrombosis (49). Importantly, PAR1 is also expressed on leukocytes and endothelial cells. Thrombin cleaves PAR1 at arginine 41 (R41) (50), generating a tethered ligand that binds to a conserved sequence in extracellular loop 2 (51). This enables the C-terminus to engage with Gαq and Gα_12/13_ which leads to inflammation, endothelial barrier permeability, and cytotoxicity (52, 53) (Figure 2). Thrombin/PAR1 activation of Gαq and Gα_12/13_ upregulates inflammatory cytokines [interleukin-1 (IL-1), IL-6, and tumor necrosis factor α (TNFα)] and endothelial adhesion molecules [E-selectin, P-selectin, intracellular adhesion molecule 1 (ICAM-1) and vascular cell adhesion molecule 1 (VCAM-1)] via activation of MAPK and NFκB. G-protein mediated Ca^2+^ signaling promotes the release of Weibel Palade bodies to the endothelial surface, increasing P-selectin and von Willebrand Factor (VWF) release, thus increasing adhesion. These signaling pathways also cause apoptosis, through caspase activation, and endothelial barrier permeability, by modulating the cytoskeleton and disrupting tight junctions (54). Thrombin/PAR1 signaling is rapid, transient, and irreversible, due to the proteolytic cleavage of the protein. Signal termination is mediated by endocytosis of the receptor in clathrin-coated pits, and lysosomal degradation (55). Matrix metalloproteases MMP-1 and MMP-13 also cleave PAR1 at aspartate 39 (D39) and serine 42 (S42), respectively, with signaling outputs similar to thrombin (53).

Interestingly, PAR1 is also cleaved by activated protein C (APC), although with lower affinity than thrombin (56). The zymogen protein C binds to endothelial protein C receptor (EPCR), a type I transmembrane protein that binds the Gla domain of protein C and APC (57). On endothelial cells, the majority of EPCR is found in caveolin-1 (Cav1)-positive lipid rafts, where it colocalizes with PAR1 (58), which is required for cytoprotective signaling (59, 60). When bound to EPCR, protein C is cleaved by thrombomodulin-bound thrombin to generate the active serine protease APC, which cleaves PAR1 at Arg46 and activates multiple signaling pathways. APC/PAR1 signaling recruits and phosphorylates β arrestin-2, which activates Rac1, inhibits NFκB, and increases the barrier integrity of the endothelium (59). β arrestin-2 also activates sphingosine kinase 1 (SphK1), which converts the lipid messenger sphingosine into sphingosine-1-phosphate (S1P). In turn, S1P activates Sphingosine-1-phosphate receptor-1 (S1PR1), which signals through Gi/Akt and disheveled 2 (Dvl2) for anti-apoptotic and anti-inflammatory signaling (61, 62). aPC/PAR1-induced β-arrestin 2 also phosphorylates ERK1/2 (61). APC thus promotes anti-inflammatory and cytoprotective signaling on ECs and preserves endothelial barrier integrity (60, 63–65) (Figure 2). APC’s cytoprotective and anti-inflammatory activity is not limited to ECs, as it can also modulate activation of monocytes, macrophages, and neutrophils, but has no effect on platelets (66). It is expressed on leukocytes (67–69), keratinocytes (70), vascular smooth muscle cells (71), cardiomyocytes (72), and neurons (73, 74), The opposing effects of PAR1 activation by thrombin and APC is a classic example of biased agonist signaling (65, 75), well described for other G-protein-coupled receptors including the neurokinin 1, angiotensin II type 1A, parathyroid hormone 1, μ opioid, and D2 dopamine receptors (76).

### The dysregulated protein C system in SCA

APC is an important natural anticoagulant (77), in addition to its critical anti-inflammatory and cytoprotective role on the endothelium (78). Zymogen protein C is a glycoprotein produced by the liver. When activated by thrombin to its serine protease form APC, it irreversibly inactivates FVa and FVIIIa by proteolysis at arginine residues (79). Anticoagulant APC activity requires its cofactor protein S, a glycoprotein that binds negatively charged phospholipid membranes via its Gla domain (78). Dysfunction or deficiencies in the protein C—protein S system are associated with venous thrombosis (80–82). It is well-documented that individuals with SCD have deficiencies in both the antigen and activity levels of protein C and protein S (29, 83–89), and that they are further decreased during crisis (90). Protein C and protein S levels negatively correlate with markers of coagulation activation (88). Although deficiencies in this system have not been linked to VOC (29), lower levels of protein C and S are associated with a higher incidence of stroke in children and adolescents with SCA (87, 89, 91, 92). The low levels of protein C and S are likely caused by multiple factors common in SCA, including decreased synthesis due to liver disease (93), consumption due to chronic activation of coagulation, and binding to phosphatidylserine-positive sickled RBCs (94).

Decreased EPCR expression and EPCR shedding occurs in inflammatory bowel disease (95), malaria (96), diabetes (97), lupus, cardiovascular ischemia–reperfusion injury (98), and endotoxemia (99). EPCR shedding is mediated by pro-inflammatory cytokines and proteases such as TNFα converting enzyme (TACE), A Disintegrin and Metalloproteinase-10 (ADAM-10) and ADAM-17. Interestingly, EPCR shedding has been observed in individuals and mice with SCD (100, 101), and EPCR-positive microparticles are found in the circulation of individuals with SCD (90). A recent abstract described loss of EPCR expression in the kidney vasculature and presence of soluble EPCR in the urine of aged sickle mice, a phenomenon that could also be triggered in young sickle mice by infusion of a low dose of heme to mimic an acute sickling event (102).

Together, these observations describe dysfunction in regulation of the vascular endothelium. In SCD, the decreased availability of the natural anticoagulant APC and its cofactor protein S, along with diminished presence of endothelial EPCR could result in reduced cytoprotective APC/PAR1 signaling. Moreover, in a disease setting characterized by chronic thrombin generation, this imbalance might favor detrimental thrombin/PAR1 signaling. This imbalance could contribute to endothelial barrier dysfunction and vascular inflammation in SCD.

### The role of PAR1 in SCA

The endothelium is chronically activated in SCA due to hemolysis (103), which contributes to the pathogenesis of vaso-occlusive events (VOE). Increased expression of adhesion molecules P-selectin and E-selectin on the endothelial surface promotes interaction with P-selectin glycoprotein ligand-1 (PSGL-1) and Cd11b/Cd18 (Mac1) on leukocytes, respectively (104). This event recruits sRBCs and platelets to form multicellular aggregates that drive vascular stasis and ultimately occlusion (105, 106). *In vitro* studies have demonstrated that activation of PAR1 with either thrombin or PAR1 agonist peptide drives the interactions between sickle RBCs and endothelial cells. This was found to be dependent on the release of P-selectin and von Willebrand Factor (VWF) from endothelial Weibel-Palade bodies (107, 108). Infusion of PAR1 agonist peptide caused rolling adhesion of sRBCs to the vascular endothelium that was p-selectin dependent in sickle mice, suggesting a role of PAR1 in vascular stasis (109). We also investigated the thrombin/PAR1 axis in sickle mice at steady state. To determine the role of PAR1, we transplanted sickle bone marrow (BM^SS^) in PAR1^−/−^ mice. Endothelial PAR1 deficiency did not affect the increased levels of thrombin generation (thrombin anti-thrombin complexes, TAT), systemic inflammation (IL-6), endothelial activation (sVCAM) or neutrophil recruitment in the lung vasculature. Interestingly thrombin inhibition with dabigatran reduced TAT, IL-6 and neutrophil recruitment to the organs (39). Agreeing with these results, Arumugam and colleagues found that decreasing expression of prothrombin reduces inflammation, vascular congestion, and improves survival of sickle mice (40). One possible interpretation of these data is that thrombin plays a role in endothelial activation and inflammation in SCD independent of PAR1, at least at steady state. An alternative hypothesis is that in BM^SS^ PAR1^−/−^ mice, the lack of PAR1 also prevents beneficial APC/PAR1 signaling. Indeed, it has been shown that administration of APC to sickle mice can attenuate thrombus formation in the cerebral microvasculature (101), indicating that APC is beneficial in SCD.

Since it is known that thrombin/PAR1 signaling contributes to endothelial P-selectin expression and sickle RBC adhesion, we also evaluated the role of this pathway in microvascular stasis. Using a dorsal skinfold chamber to evaluate blood flow in the skin microvasculature, we found that inhibition of PAR1 with the irreversible orthosteric antagonist vorapaxar protected sickle mice from heme-induced microvascular stasis (43). Similar results were obtained in BM^SS^ PAR1^−/−^ mice, which also had significantly less endothelial P-selectin and VWF expression in lung tissue after heme treatment (43). These data suggest that thrombin/PAR1 activation might play a role in the cell–cell interactions that lead to VOC (43, 109), and that APC/PAR1 signaling can be beneficial (101), in SCD. Future studies should be aimed at determining the role of PAR1 in other acute and chronic complications of SCD, including stroke, thrombosis, and acute chest syndrome.

### Current therapeutic strategies to target PAR1

Most PAR1 antagonists were designed to attenuate thrombin-mediated platelet activation and reduce thrombosis. Vorapaxar is an orally available antagonist that binds the extracellular pocket of PAR1 irreversibly and with high affinity. It effectively blocks PAR1 activation by both thrombin and APC. In clinical trials, administration of vorapaxar in combination with dual antiplatelet therapy improved cardiovascular outcomes but increased the risk of bleeding, especially in patients with a history of stroke (110). Thus, its use is counter-indicated in SCD patients. Recombinant APC (Xigris) was tested in pre-clinical models of sepsis, but had limited success and also increased the risk of bleeding in larger clinical trials (45). A signaling-selective variant of APC with limited anti-coagulant activity, 3K3A-APC, is also being tested for the treatment of stroke and amyotrophic lateral sclerosis (NCT02222714 and NCT05039268).

Another option for targeting PAR1 are small molecules. Q94 is an allosteric modulator that is thought to act at the intracellular face of PAR1. Although it inhibits PAR1-dependent platelet activation, it has limited efficacy on endothelial PAR1 signaling (111). Pepducins are a family of PAR1 modulators; they are biomimetic lipidated peptides that can enter the cell and target the intracellular loops of a receptor (112–114). One pepducin, PZ-128, is currently being tested in clinical trials for coronary artery disease (114) and has a promising safety profile. Parmodulins are small molecule allosteric modulators of PAR1, which bind the intracellular C-terminus and recruit β arrestin (115). They not only block thrombin-dependent PAR1 signaling; they can actually induce APC-like cytoprotective and anti-inflammatory signaling. Parmodulins have been shown to have significant anti-thrombotic and anti-inflammatory effects in mouse models of venous thrombosis (116, 117), neurologic diseases (115), virus (118), and diabetes (119).

## Conclusion

The APC-EPCR-PAR1 axis is plays an important role in maintaining vascular endothelial homeostasis, and several studies described herein suggest that this pathway is dysfunctional in SCA. We speculate that chronic thrombin generation which can activate detrimental PAR1 signaling, paired with decreased APC/PAR1 signaling due to APC consumption and EPCR shedding, might play a role in the activated vascular endothelium in SCD.

## Author contributions

NR and ES wrote and edited the manuscript. All authors contributed to the article and approved the submitted version.

## Funding

This work was supported by NHLBI R01HL155193.

## Conflict of interest

The authors declare that the research was conducted in the absence of any commercial or financial relationships that could be construed as a potential conflict of interest.

## Publisher’s note

All claims expressed in this article are solely those of the authors and do not necessarily represent those of their affiliated organizations, or those of the publisher, the editors and the reviewers. Any product that may be evaluated in this article, or claim that may be made by its manufacturer, is not guaranteed or endorsed by the publisher.
